# A SARS-CoV-2 spike ferritin nanoparticle vaccine protects hamsters against Alpha and Beta virus variant challenge

**DOI:** 10.1038/s41541-021-00392-7

**Published:** 2021-10-28

**Authors:** Kathryn McGuckin Wuertz, Erica K. Barkei, Wei-Hung Chen, Elizabeth J. Martinez, Ines Lakhal-Naouar, Linda L. Jagodzinski, Dominic Paquin-Proulx, Gregory D. Gromowski, Isabella Swafford, Akshaya Ganesh, Ming Dong, Xiankun Zeng, Paul V. Thomas, Rajeshwer S. Sankhala, Agnes Hajduczki, Caroline E. Peterson, Caitlin Kuklis, Sandrine Soman, Lindsay Wieczorek, Michelle Zemil, Alexander Anderson, Janice Darden, Heather Hernandez, Hannah Grove, Vincent Dussupt, Holly Hack, Rafael de la Barrera, Stasya Zarling, James F. Wood, Jeffrey W. Froude, Matthew Gagne, Amy R. Henry, Elham Bayat Mokhtari, Prakriti Mudvari, Shelly J. Krebs, Andrew S. Pekosz, Jeffrey R. Currier, Swagata Kar, Maciel Porto, Adrienne Winn, Kamil Radzyminski, Mark G. Lewis, Sandhya Vasan, Mehul Suthar, Victoria R. Polonis, Gary R. Matyas, Eli A. Boritz, Daniel C. Douek, Robert A. Seder, Sharon P. Daye, Mangala Rao, Sheila A. Peel, M. Gordon Joyce, Diane L. Bolton, Nelson L. Michael, Kayvon Modjarrad

**Affiliations:** 1 U.S. Military HIV Research Program, Center for Infectious Diseases Research, Walter Reed Army Institute of Research, Silver Spring, MD USA; 2 Veterinary Pathology Division, Walter Reed Army Institute of Research, Silver Spring, MD USA; 3 Emerging Infectious Diseases Branch, Center for Infectious Diseases Research, Walter Reed Army Institute of Research, Silver Spring, MD USA; 4 Henry M. Jackson Foundation for the Advancement of Military Medicine, Bethesda, MD USA; 5 Diagnostics Countermeasures Branch, Center for Infectious Diseases Research, Walter Reed Army Institute of Research, Silver Spring, MD USA; 6 Virus Diseases Branch, Center for Infectious Diseases Research, Walter Reed Army Institute of Research, Silver Spring, MD USA; 7 Oak Ridge Institute of Science and Education, Oak Ridge, TN USA; 8 Pathology Division, United States Army Medical Research Institute of Infectious Diseases, Fort Detrick, Frederick, MD USA; 9 Pilot Bioproduction Facility, Walter Reed Army Institute of Research, Silver Spring, MD USA; 10 Human Immunology Section, Vaccine Research Center, National Institute of Allergy and Infectious Diseases, National Institutes of Health, Bethesda, MD USA; 11 Virus Persistence and Dynamics Section, Vaccine Research Center, National Institute of Allergy and Infectious Diseases, National Institutes of Health, Bethesda, MD USA; 12 W. Harry Feinstone Department of Molecular Microbiology and Immunology, Johns Hopkins Bloomberg School of Public Health, Baltimore, MD USA; 13 BioQual, Inc., Rockville, MD USA; 14 Emory Vaccine Center, Department of Pediatrics, Emory School of Medicine, Atlanta, GA USA; 15 Cellular Immunology Section, Vaccine Research Center, National Institute of Allergy and Infectious Diseases, National Institutes of Health, Bethesda, MD USA; 16 One Health Branch, Walter Reed Army Institute of Research, Silver Spring, MD USA; 17 Center for Infectious Diseases Research, Walter Reed Army Institute of Research, Silver Spring, MD USA

**Keywords:** SARS-CoV-2, Protein vaccines, Viral infection

## Abstract

The emergence of SARS-CoV-2 variants of concern (VOC) requires adequate coverage of vaccine protection. We evaluated whether a SARS-CoV-2 spike ferritin nanoparticle vaccine (SpFN), adjuvanted with the Army Liposomal Formulation QS21 (ALFQ), conferred protection against the Alpha (B.1.1.7), and Beta (B.1.351) VOCs in Syrian golden hamsters. SpFN-ALFQ was administered as either single or double-vaccination (0 and 4 week) regimens, using a high (10 μg) or low (0.2 μg) dose. Animals were intranasally challenged at week 11. Binding antibody responses were comparable between high- and low-dose groups. Neutralizing antibody titers were equivalent against WA1, B.1.1.7, and B.1.351 variants following two high dose vaccinations. Dose-dependent SpFN-ALFQ vaccination protected against SARS-CoV-2-induced disease and viral replication following intranasal B.1.1.7 or B.1.351 challenge, as evidenced by reduced weight loss, lung pathology, and lung and nasal turbinate viral burden. These data support the development of SpFN-ALFQ as a broadly protective, next-generation SARS-CoV-2 vaccine.

## Introduction

Since the beginning of the COVID-19 pandemic, an unprecedented global effort has resulted in the rapid emergence, and early regulatory authorization or approval, of multiple vaccines for use in humans^1^. The emergence of variants of concern (VOC) underscores the need for continued development of next-generation vaccines^2,3^. Increasingly, VOCs are becoming the dominant circulating lineages world-wide, owing to their increased transmissibility and potential to cause breakthrough infection in vaccinated individuals. Two VOCs have gained particular attention: B.1.1.7 and B.1.351, first detected in the United Kingdom and in the Republic of South Africa, respectively^4–10^. Recent studies have shown reduced cross-neutralizing antibody responses from convalescent and vaccinee sera against both VOCs, but B.1.351 in particular^4,5,8,11,12^. Consequently, there has been a renewed global effort to develop new SARS-CoV-2 vaccines, that effectively confer protection against current and emerging SARS-CoV-2 VOCs^4,13,14^.

We recently developed a novel SARS-CoV-2 vaccine that presents eight prefusion-stabilized spike glycoprotein trimers in an ordered array on a ferritin nanoparticle and is adjuvanted with Army Liposomal Formulation QS21 (SpFN-ALFQ). This vaccine formulation has demonstrated efficacy against an early isolate of the origin SARS-CoV-2 variant (WA1) in both K18 murine and rhesus macaque viral challenge models^15–18^. In rhesus macaques, SpFN-ALFQ elicited a dose-dependent potent humoral and cellular immune response that translated into a precipitous reduction in viral load upper and lower airways of the animals, as well as protection from lung histopathology. Importantly, immunization induced cross-neutralizing antibodies against current circulating VOCs^16^. Building on these data, we evaluated the efficacy of SpFN-ALFQ to protect against virus challenge with VOCs B.1.1.7 and B.1.351 in a Syrian golden hamster (SGH) challenge model. This animal model has become a standard in the field for pre-clinical SARS-CoV-2 vaccine development, as respiratory pathology in this model closely recapitulates human disease^19–22^.

Here, we demonstrate that SpFN-ALFQ generates strong binding antibody responses against the receptor-binding domain and spike proteins of both B.1.1.7 and B.1.351, as well as potent neutralizing antibody responses against both VOCs. Consistent with development of a protective humoral response, we demonstrate that SpFN-ALFQ confers clear protection against upper respiratory tract disease following challenge with these VOCs, as demonstrated by less body weight loss and decreased tissue viral burden and lung pathology. The SpFN-ALFQ vaccine candidate is now currently under assessment in a human phase 1 clinical trial (ClinicalTrials.gov Identifier: NCT04784767). These data support further development of the vaccine as one that may be broadly applicable to multiple sarbecovirus lineages.

## Results

### Hamster model to assess efficacy of SpFN-ALFQ against VOCs B.1.1.7 and B.1.351

**Fig. 1:**
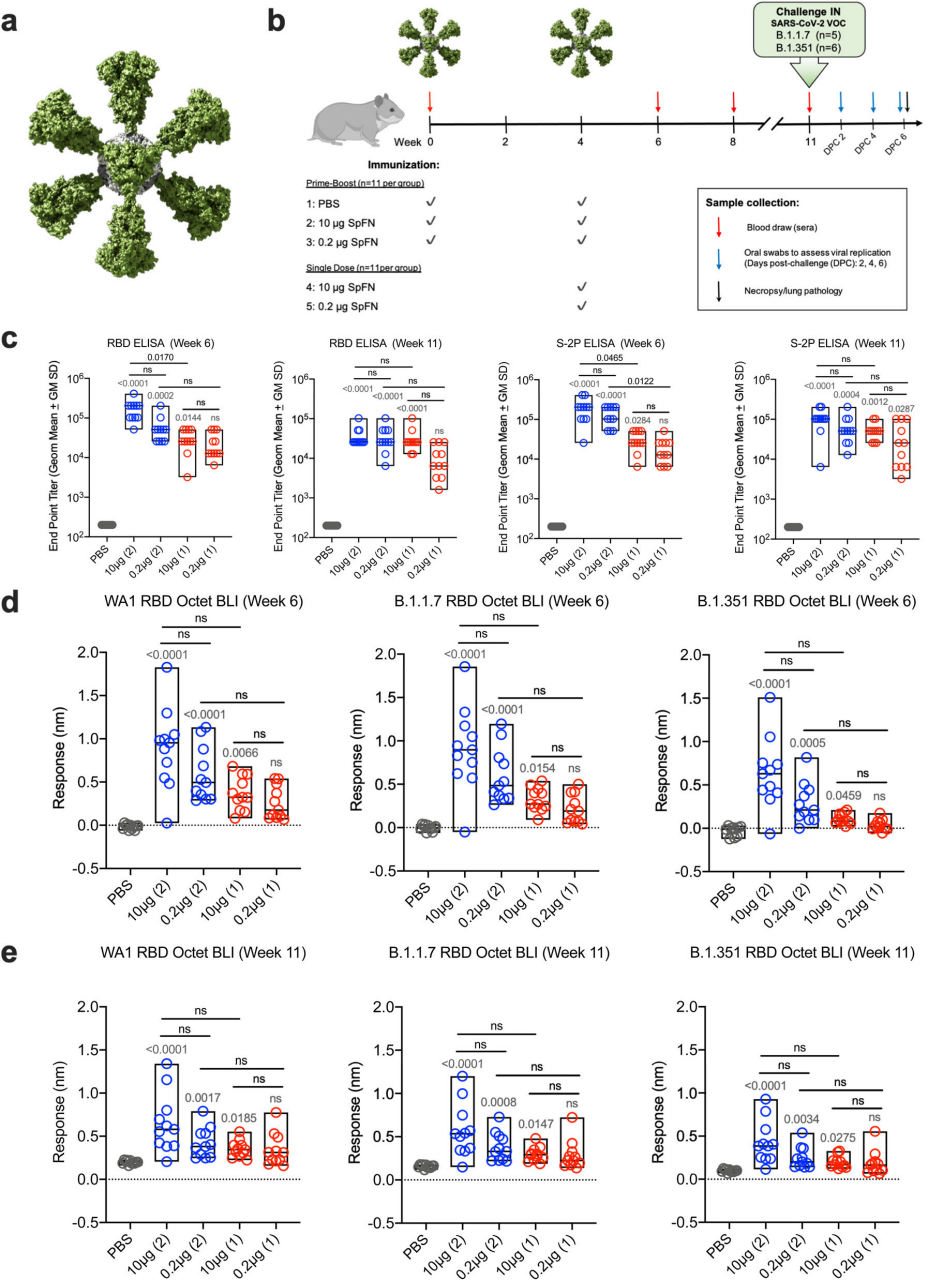
Antibody responses following SpFN-ALFQ immunization. **a** The 3-dimensional model of SpFN with Spike protein trimers (green) decorating a ferritin core (gray) as viewed down a 3-fold axis. **b** Experimental design with hamsters receiving immunization at weeks 0 and 4 in the 2-dose regimen and week 4 in the 1-dose regimen as depicted by green SpFN structures above the time-line and check marks below the timeline indicating immunogen dose (10 μg or 0.2 μg or PBS control). Phlebotomy samples were taken at weeks 0, 6, 8, and 11 as indicated by red arrows. Intranasal (IN) viral challenge was performed at week 11 with either VOC B.1.1.7 or B.1.351 viral stocks with the number of animals challenged noted parenthetically. Oral swabs were collected at 2, 4, and 6 days post challenge (DPC) as indicated by blue arrows above the timeline. Necropsy was performed on all animals at day 6 post-challenge as indicated by the black arrow. **c** ELISA was performed using either WA1 derived Receptor Binding Domain (RBD) or S-2P Spike proteins from sera taken at weeks 6 and 11. Sera from week 0 was also assessed, with no detectable signal observed in any samples (data not shown). The vaccine regimens are indicated on the *x*-axis by PBS (control) or SpFN concentration with the number of vaccinations in the regimen given parenthetically and by color code (blue, 2-dose, red, 1-dose). Endpoint titers are graphed as geometric mean titers. **d**, **e** Octet Biolayer Interferometry (BLI) responses against the WA1, B.1.1.7, and B.1.351 sequences of the RBD are given for the vaccination regimens as in c at weeks 6 (**d**) and 11 (**e**). BLI responses are given in nanometers (nm) on the *y*-axis. Box plot bounds depict the standard deviation and the center line the median value. *p*-values for active vaccination groups compared with PBS control are given just above the boxes in light gray; intra-active regimen *p*-values are given above the boxes in black. ns not significant (*p* > 0.05) using the Kruskal–Wallis multiple comparisons test, with Dunn’s correction.

Prior studies have demonstrated that the SpFN-ALFQ vaccine is efficacious against the WA1 strain of SARS-CoV-2 in a nonhuman primate model^16^. The ability of this vaccine to confer protection against the VOC B.1.1.7, now prominent throughout the U.S. and other parts of the world, and B.1.351, prominent in Africa and emerging world-wide, is unknown. We evaluated vaccine immunogenicity and efficacy against VOCs in SGH due to the susceptibility of SGH to severe clinical disease, lung pathology, and viral replication in the respiratory tract^22,23^. SpFN (Fig. 1a) was administered at a high (10 μg) or low-dose (0.2 μg) adjuvanted with a fixed ALFQ concentration, selected based on immunogenicity and efficacy studies of SpFN-ALFQ in mice^18^, in a either a single (1) and two (2)-vaccination regimen in parallel with 2 injections of phosphate buffered saline (PBS) in control animals (Fig. 1b). Blood for serologic analysis was drawn periodically throughout the study, with key immunogenicity timepoints at weeks 6 and 8 (two and four weeks following last vaccine dose, respectively) and week 11 (seven weeks post final vaccine dose), preceding viral challenge.

### Binding antibody responses elicited by two-dose SpFN-ALFQ vaccination

Robust serum binding antibody responses to the SARS-CoV-2 WA1 spike protein (S-2P) and receptor-binding domain (RBD) were observed at week 6 by enzyme linked immunosorbent assay (ELISA) (Fig. 1c). Both the 1- and 2-dose regimens were immunogenic, with higher ELISA titers elicited by the 2-dose regimen against both RBD and S-2P. We did not observe significant differences between high and low doses, within either the 1- and 2-dose regimens. Endpoint titers against S-2P at week 6 were significantly different within the 10 μg and 0.2 μg groups between dosing regimens, with mean reciprocal dilutions for the 1-dose regimens of 3.2 × 10^4^ (10 μg) and 1.92 × 10^4^ (0.2 μg), and 2-dose regimen responses of 1.98 × 10^5^ (10 μg) and 1.35 × 10^5^ (0.2 μg). Endpoint titers did not substantially wane between weeks 6 (Fig. 1c) and 8 (Supplementary Fig. 1a) or week 11 (Fig. 1c), indicating sustainment of binding antibody responses.

We then assessed the breadth of post-vaccination binding antibody responses to WA1 and VOCs B.1.1.7 and B.1.351 variant RBD antigens, measured by biolayer interferometry (Fig. 1d). Responses again trended higher with the 2-dose regimen and did not differ between the 10 μg and 0.2 μg groups within regimens. Binding levels between strains were comparable between WA1 and B.1.1.7 in the 2-dose regimens with B.1.1.7/WA1 mean fold change ratios of 1.02 (10 μg) and 1.01 (0.2 μg), while 1-dose regimens of B.1.1.7/WA1 ratios were 0.88 (10 μg) and 0.82 (0.2 μg). Comparatively, B.1.351 cross-reactive binding antibody levels were reduced compared to WA1. B.1.351/WA1 mean fold-change ratios in the 2-dose regimens were 0.71 (10 μg) and 0.49 (0.2 μg), and in the 1-dose regimens 0.29 (10 μg) and 0.14 (0.2 μg). Similar magnitude responses were observed at week 8 (Supplementary Fig. 1b), while binding antibody levels declined by week 11, the time of challenge (Fig. 1e). Cross-reactive binding antibodies at week 6 also recognized heterologous B.1.1.7 and B.1.351 cell surface expressed spike protein in a flow cytometric based IgG opsonization assay; responses were consistent with the quantitative differences seen for the inter-group comparisons in Fig. 1 for all three strains (Supplementary Fig. 1c).

### hACE2 competition and pseudovirus neutralizing antibody responses

**Fig. 2:**
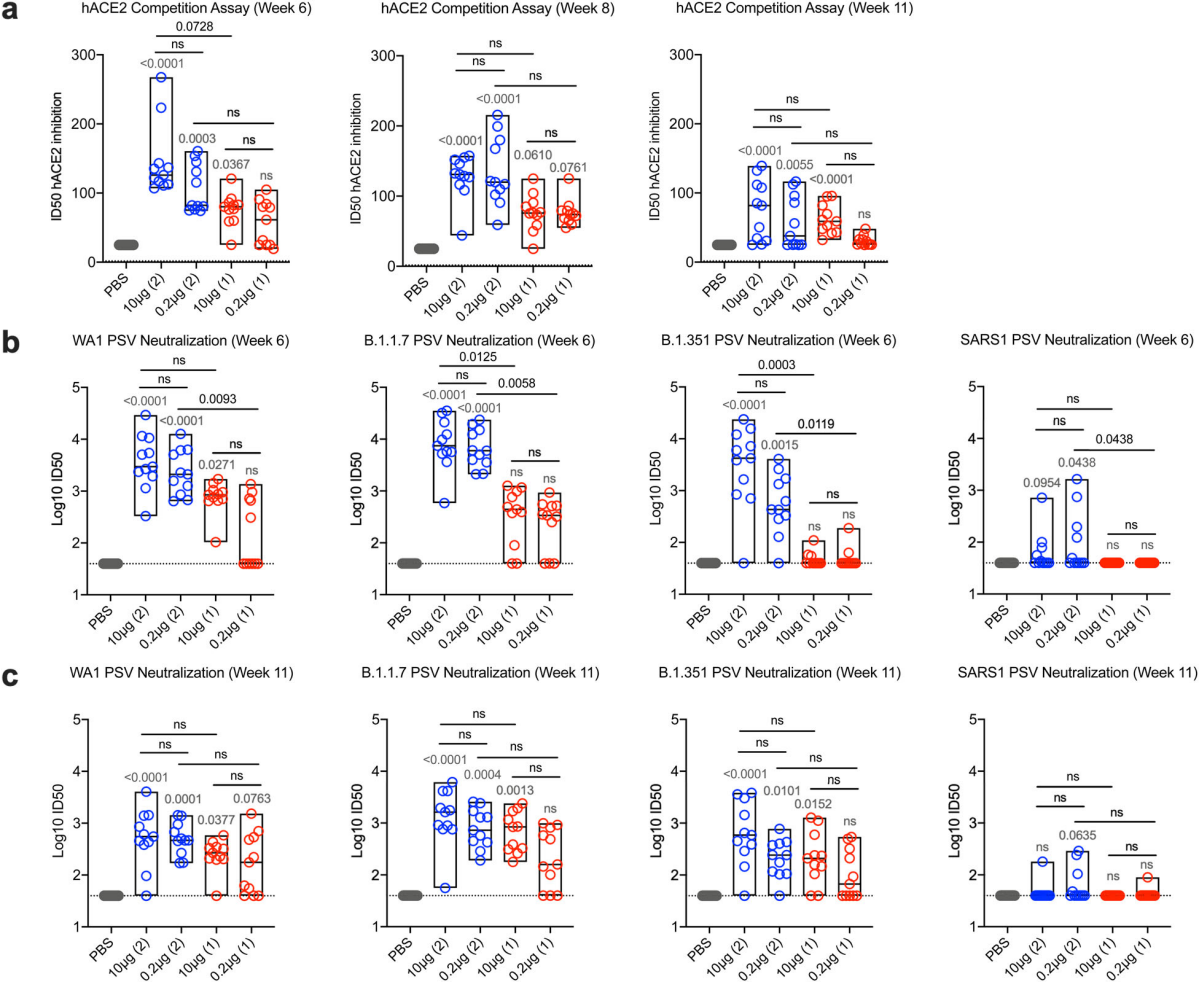
Human angiotensin-converting enzyme competition and pseudovirus neutralization responses following SpFN-ALFQ immunization. **a** Human angiotensin-converting enzyme competition (hACE2) assays were performed from sera taken at week 6, 8, and 11. The vaccine regimens are indicated on the *x*-axis by PBS (control) or SpFN-ALFQ concentration with the number of vaccinations in the regimen given parenthetically and by color code (blue, 2-dose, red, 1-dose). Inhibitory dose 50% (ID50) are given on the *y*-axis. **b**, **c** Pseudovirus neutralization at weeks 6 and 11 are given against Spike proteins derived from WA1, B.1.1.7, B.1.351 SARS-CoV-2 variants and from SARS-CoV-1. The neutralization titers are given as inhibitory dose 50% (ID50) on the *y*-axis in logarithmic scale. Box plot bounds depict the standard deviation and the center line the median value. *p*-values for SpFN-ALFQ vaccination groups compared with PBS control are given just above the boxes in light gray while intra-active regimen *p*-values are given above the boxes in black. ns not significant (*p* > 0.05) using the Kruskal–Wallis multiple comparisons test, with Dunn’s correction.

As SARS-CoV-2 mediates host cell entry via engagement of the viral spike protein with cell surface expressed human angiotensin-converting enzyme 2 (hACE2) protein, preventing this interaction is a critical target for vaccine-induced immune responses^24^. We measured hACE2 inhibitory antibodies longitudinally using a serum competition assay that detects blockade of RBD-hACE2 binding (Fig. 2a). At week 6, mean 50% inhibitory dilution (ID_50_) values in the 2-dose regimens were 145.6 (10 μg) and 106.8 (0.2 μg), and 76.6 (10 μg) and 57.0 (0.2 μg) in the 1-dose regimens. Responses did not differ within regimens and were largely stable at week 8 but waned by the time of challenge at week 11.

Pseudovirus neutralizing antibody responses were measured against the WA1, B.1.1.7, and B.1.351 variants at weeks 6 and 11 (Fig. 2b, c, respectively). At week 6, responses were higher for the 2-dose versus the 1-dose vaccine regimen in most cases (Fig. 2b). Mean log10 ID_50_ values against WA1 ranged from 3.56 (10 μg) to 3.36 (0.2 μg) and 2.88 (10 μg) to 2.17 (0.2 μg) to WA1 in the 2-dose and 1-dose regimens, respectively, with comparable titers against B.1.1.7 of 3.90 (10 μg), 3.82 (0.2 μg), 2.52 (10 μg), and 2.36 (0.2 μg), respectively. Responses to B.1.351 were diminished relative to WA1 and B.1.1.7, notably with the 1-dose regimen, with mean log10 ID_50_ values of 3.46 (10 μg)–2.74 (0.2 μg) and 1.67 (10 μg)–1.68 (0.2 μg) in the 2-dose and 1-dose regimens, respectively. Titers were consistently higher for the 2-dose versus the 1-dose regimens. Neutralizing antibody responses decreased between weeks 6 and 11 and the differences between the 2-dose and 1-dose regimens were less marked (Fig. 2c). Interestingly, cross-neutralization was observed for some animals in the 2-dose regimen against the related sarbecovirus SARS-CoV-1, albeit at reduced levels compared to SARS-CoV-2. Here we demonstrate that SpFN-ALFQ vaccine induces potent neutralization against SARS-CoV-2 VOCs, as well as cross-neutralization against SARS-CoV-1, as previously described in rhesus macaques and mice^16–18^.

### Determination of body-weight loss in SpFN-ALFQ vaccinated, VOCs challenged hamsters

**Fig. 3:**
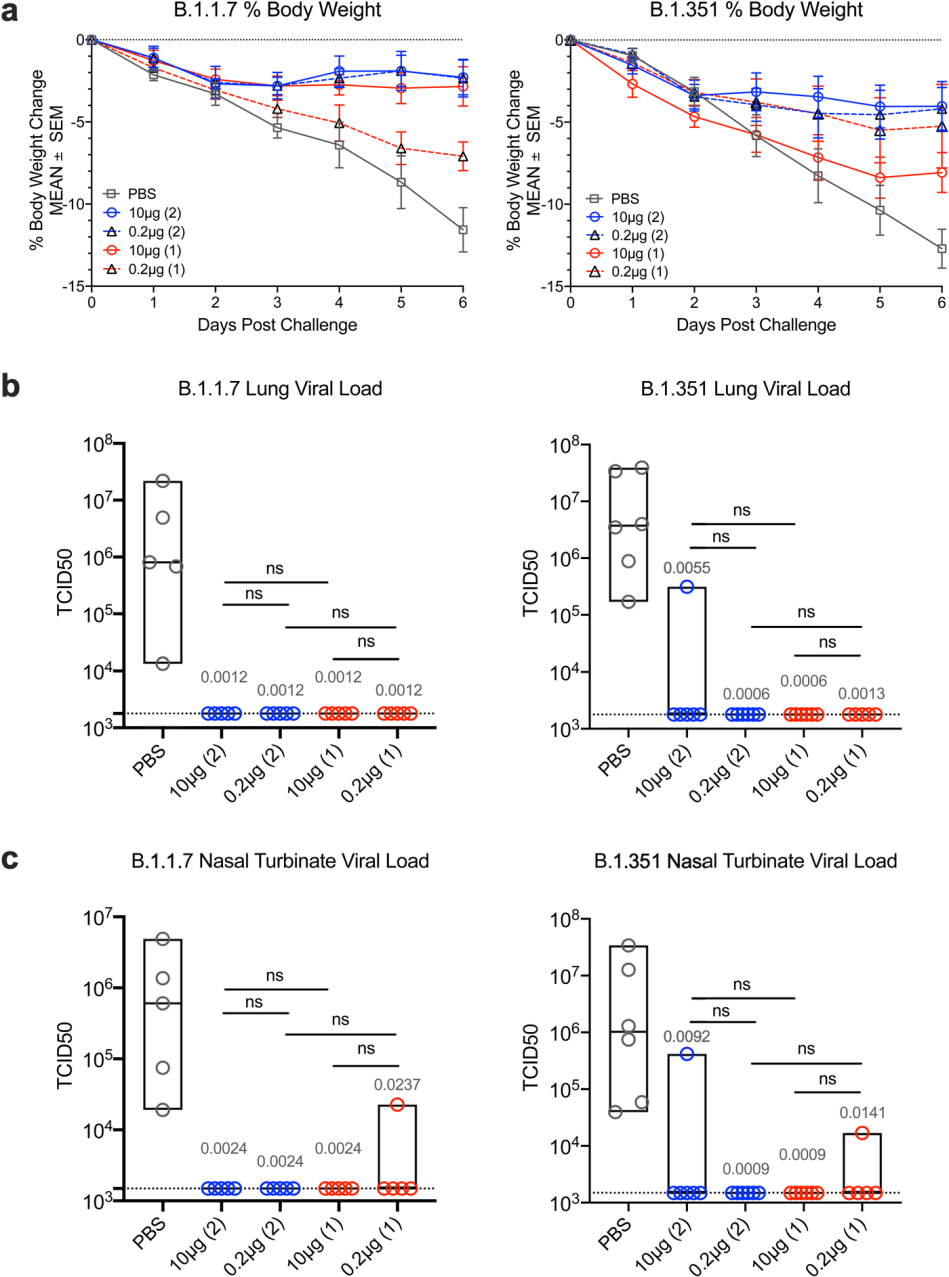
Body weight changes and lung viral load post-challenge. Daily weights were gathered on hamsters from the time of viral challenge to necropsy on day 6 post challenge when lungs were harvested for a whole tissue, culture based viral load assessment in Vero TMPRSS2 cells. **a** Mean percent body weight changes plus and minus standard error of the mean (SEM) are given on the *y*-axis for groups of hamsters assigned to phosphate buffered saline control (PBS) or SpFN-ALFQ vaccination from day of challenge (day 0) to day of necropsy (day 6) for either B.1.1.7 or B.1.351 challenge. Data from immunization groups are given in each graph as PBS (gray plot, phosphate buffered saline control) or active immunogen dose (blue circle/ blue solid line, 10 μg 2-dose regimen; black triangle/ blue dotted line, 0.2 μg 2-dose regimen; red circle/red solid line, 10 μg 1-dose regimen; black triangle/red dotted line, 0.2 μg 1-dose regimen. Number of vaccinations in the vaccine regimen are also given parenthetically within each graph. **b** SARS-CoV-2 viral load data from lung tissue harvested on day 6 post challenge is given on the *y*-axis as the tissue culture infective dose, 50% (TCID50) per gram of tissue as titered on Vero TMPRSS2 cells and read out by cytopathic effects for either B.1.1.7 and B.1.351 challenged hamsters. Immunization groups are given on the x-axis as PBS (control (gray circles); 10 μg and 0.2 μg 2-dose vaccine regimens (blue circles); 10 μg and 0.2 μg 1-dose vaccine regimens (red circles). **c** SARS-CoV-2 viral load data from nasal turbinate tissue harvested on day 6 post challenge is given as in **b**. Group data are displayed as box plots with the top and bottom bars of the box the standard deviation and the center line as median group values. The dotted horizontal line is the lower limit of detection of the assay. *p*-values for SpFN-ALFQ vaccination groups compared with PBS control are given just above the boxes in light gray while intra-active regimen *p*-values are given above the boxes in black. ns not significant (*p* > 0.05) using the Kruskal–Wallis multiple comparisons test, with Dunn’s correction.

Hamsters were divided into two cohorts comprised of male and female animals and challenged with either B.1.1.7 (*n* = 5) or B.1.351 (*n* = 6) variants of SARS-CoV-2 by intranasal inoculation at week 11, 7 weeks after the last immunization in either the 2-dose or 1-dose regimen groups. Concentration of viral challenge doses were selected based on prior titration of viral stocks in hamsters, targeting a 10–15% loss of body weight by 7 days post-challenge. Body weight was monitored daily following challenge. For both B.1.1.7 and B.1.351 infection, weight loss of PBS control animals fell as expected between 10–15% by 6 days post-challenge (DPC), with a mean weight loss of 11.6% for B.1.1.7 and 12.7% for B.1.351 respectively (Fig. 3a). For the 10 μg 2-dose regimen, B.1.1.7 challenged animals had a mean body weight loss of 2.3% (9.3% difference compared to PBS control) and B.1.351 challenged animals with slightly higher at 4.0% (8.7% difference compared to PBS control), but still a dramatic reduction from the PBS vaccinated control hamsters. In the 2-dose regimen, both the 10 μg and 0.2 μg dose groups demonstrated comparable levels of protection, with the 1-dose vaccinated animals demonstrating the least protection from body-weight loss both for B.1.1.7 and B.1.351 challenges.

### Viral burden in lung and nasal tissues SpFN-ALFQ vaccinated, B.1.1.7 and B.1.351 challenged hamsters

To assess the impact of SpFN-ALFQ immunization following challenge by either the B.1.1.7 or B.1.351 strains, viral load was assessed by viral culture recovery in lung tissues at day 6 post challenge (Fig. 3b). The mean viral load in the PBS vaccinated control animals challenged with B.1.1.7 was 5.67 × 10^6^ TCID50/gram of lung tissue. Virus was not recovered in lung tissue culture in both the 10 and 0.2 μg 2-dose and 1-dose regimens (below the limit of detection of 1.78 × 10^3^) (Fig. 3b). The mean lung tissue viral load in the PBS control vaccinated animals challenged with B.1.351 was 1.36 × 10^7^ TCID50/g. All animals in the 2-dose and 1-dose regimens, for both 10 μg and 0.2 μg doses, showed no detectable virus except for one animal in the 10 μg 2-dose vaccinated group (Fig. 3b). Viral load was also measured in nasal turbinate tissue collected at day 6 post-challenge, with similar results to lung tissue analysis (Fig. 3c). The mean nasal turbinate tissue viral load in the PBS control vaccinated animals challenged with B.1.1.7 was 1.39 × 10^6^ TCID50/g and B.1.351 was 8.14 × 10^6^ TCID50/g of nasal turbinate, respectively. All vaccinated animals were below the limit of detection (1.492 × 10^3^ TCID50/g), except a single outlier animal in the 0.2 μg 1-dose regimen for B.1.1.7 challenged group, and an outlier animal each in the 10 μg 2-dose and 0.2 μg 1-dose regimens for B.1.351 challenged animals (Fig. 3c). Throughout the challenge phase of the study, oral swabs were collected at days 2, 4, and 6 post-challenge and assessed for viral burden by RT-qPCR, by measuring both subgenomic E messenger RNA (sgmRNA) (Supplementary Fig. 2a, c) and total viral RNA (viral load) (Supplementary Fig. 2b, d). Overall oral viral loads decreased modestly over the duration of the challenge in all groups for both B.1.1.7 and B.1.351, which may partially be impacted by residual detection of viral inoculum. These results demonstrate clear protection from tissue viral load in vaccinated animals challenged with B.1.1.7 and B.1.351, a critical determinant in establishing effective vaccines against SARS-CoV-2 VOCs.

### Impact of SpFN-ALFQ vaccination on lung histopathology and tissue viral detection by immunohistochemistry

**Fig. 4:**
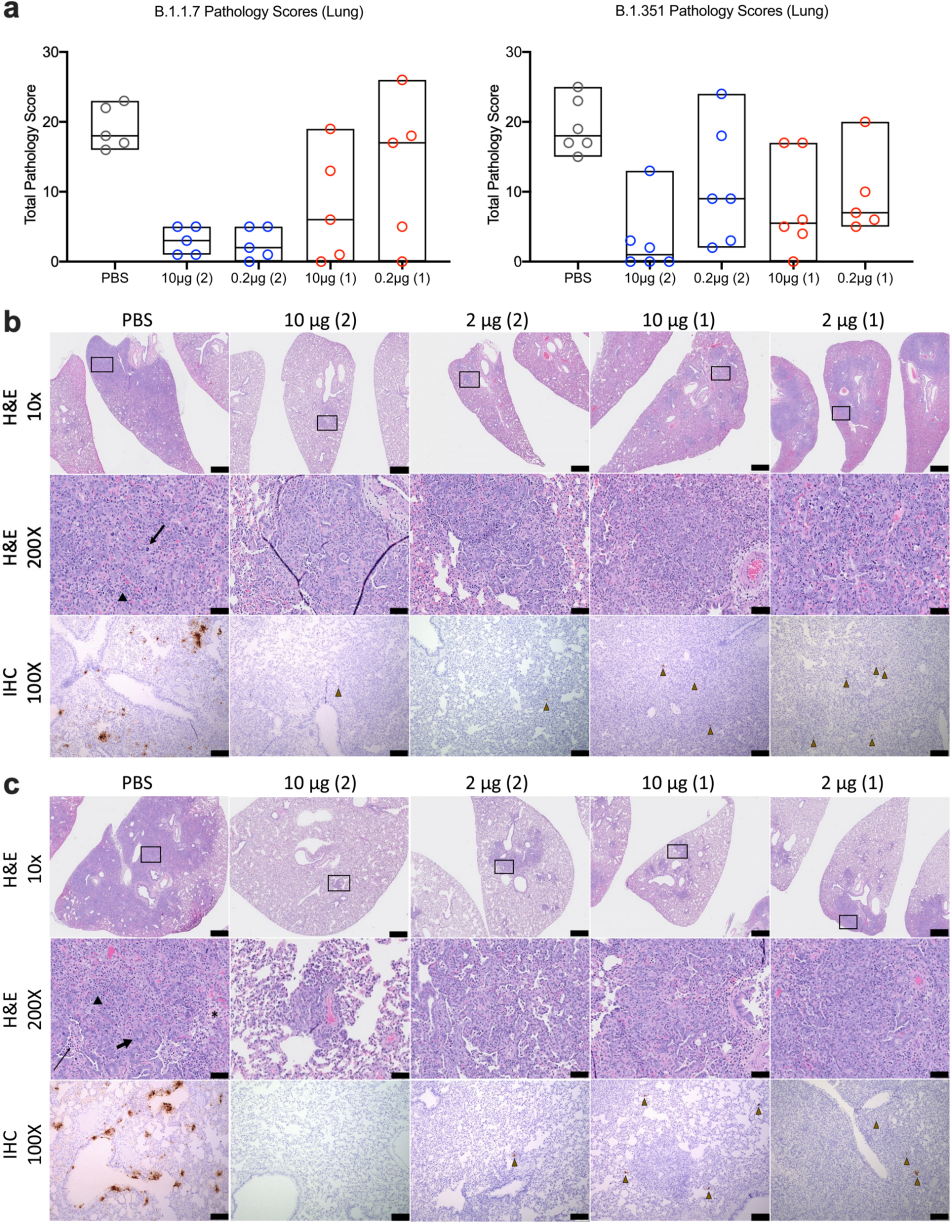
Standard and immunohistopathologic examination post-challenge. Lung tissues were collected at necropsy on day 6 post-challenge, fixed with neutral-buffered formalin, and stained with hematoxylin and eosin (H&E) for standard microscopic examination as well has submitted for immunohistochemical (IHC) staining for SARS-CoV-2 nucleocapsid (N) protein. **a** H&E stained slides were scored for pathologic effects on the *y*-axis (see Methods section) for B.1.1.7 (left) and B.1.351 (right) challenged hamsters. Vaccination group box plot bounds reflect minimum and maximum values and the center line is the median group score. Vaccination groups are given as: PBS control (gray circles); 10 μg and 0.2 μg 2-dose vaccine regimens (blue circles); 10 μg and 0.2 μg 1-dose vaccine regimens (red circles). **b**, **c** Representative lung tissue sections from the PBS control and 10 μg and 0.2 μg 2-dose and 1-dose regimens with the number of vaccinations given parenthetically for B.1.1.7 (**b**) and B.1.351 (**c**) challenged hamsters in the columns as indicated. Rows are given by either H&E at 10- and 200-times magnification power (×10 and ×200, respectively) or IHC of SARS-CoV-2 viral antigen at 100 times magnification power (×100). The black boxes in the top row indicate the area magnified in the middle row. Interstitial pneumonia is characterized by inflammatory cellular infiltrates (triangle), type II pneumocyte hyperplasia (thick arrow), bronchiolar epithelial hyperplasia, bronchiolar exudate (thin arrow), and edema (asterisk). SARS-CoV-2 immunopositive cells are highlighted by brown triangles. Scale bars: top row, 1 mm; middle row, 50 µm; bottom row, 100 µm.

Lung pathology was assessed day 6 post-challenge by both routine hematoxylin and eosin (H&E) staining as well as immunohistochemistry (IHC) for the presence of viral nucleocapsid (N) protein (Fig. 4). By semiquantitative scoring of lung histopathology, the highest degree of pathology was seen in the PBS vaccinated control animals challenged with either B.1.1.7 or B.1.351 (Fig. 4a). All PBS vaccinated control animals developed histopathologic evidence of interstitial pneumonia (IP) ranging from multifocal to extensive (distribution), moderate to marked (severity) (Fig. 4b, c, for B.1.1.7 and B.1.351 challenged animals, respectively). The pneumonia was characterized by type II pneumocyte hyperplasia, alveolar edema, alveolar inflammatory and necrotic debris, thickening of alveolar septae, bronchiolar epithelial hyperplasia, and increased numbers of pulmonary macrophages (including multinucleated giant cells). The extent of IP present in PBS vaccinated control animals was comparable in animals challenged with either B.1.1.7 or B.1.351. In the B.1.1.7 challenged animals, the least amount of pathology was seen in the animals vaccinated with either 2-dose regimen, although less pathology was evident in all of the SpFN-ALFQ vaccination groups compared with the PBS control group (Fig. 4a - left panel, b, c and Supplementary Table 1a). For the B.1.351 challenged animals, the most protection from lung pathology was observed in the 10 μg, 2-dose regimen, although there was a decrease in the mean pathology score of all SpFN-ALFQ vaccinated groups (Fig. 4a - right panel, b, c and Supplementary Table 1a). Overall, there was a trend toward increased pathology observed in the B.1.351 versus the B.1.1.7 animals, in particular within the 1-dose regimen. In the 10 μg 2-dose regimen however, protection between VOCs were comparable. Immunohistochemistry demonstrated strong, multifocal to focally extensive (>500–1000 cells per section) immunopositivity to SARS-CoV-2 nucleocapsid (N) protein in bronchiolar epithelium, alveolar pneumocytes and pulmonary macrophages in the lungs of all unvaccinated animals. Viral antigen detected in lung sections from animals in the 10 µg and 0.2 µg vaccine groups was substantially reduced compared with the PBS vaccinated groups with the greatest reduction seen in the 10 µg 2-shot vaccine groups (Supplementary Table 1b). Taken together, SpFN adjuvanted with ALFQ confers clear protection from viral burden and pathology in the lungs following VOC challenge.

## Discussion

The development of effective and safe vaccines in response to the SARS-CoV-2 pandemic has occurred at an unprecedented rate. This success has been tempered by the reduced vaccine efficacy against some emerging VOCs, most notably B.1.351^4,5,8,11,14,25^. The need for more broadly effective vaccines against multiple lineages of SARS-CoV-2 will likely increase, as underscored by the rapid rise of VOCs in India, including B.1.1.7 and emerging variants B.1.617 with derivative lineages, and B.1.618^3^. As B.1.1.7 has become the dominant variant in the U.S.^26^ and B.1.351 and B.1.617 lineages are becoming more dominant in many areas of the world, evaluation of existing and novel vaccines against these strains is essential. As the pandemic continues, SARS-CoV-2 will likely adapt not only to replicate more efficiently in the human host and evolve to escape host immune responses to natural infection, it may increasingly adapt to vaccine evoked immune responses. This large number of products administered globally, with differential vaccine efficacy, will further drive the evolution of virus variants that may be more resistant to current formulations of SARS-CoV-2 vaccines. This will be especially true in populations where immunocompromised individuals are prevalent (e.g., HIV infection, cancer therapies, organ transplant, and autoimmune diseases) providing an ideal milieu for the generation of virus variants to challenge current and next-generation SARS-CoV-2 vaccines^27^.

We evaluated the efficacy of a novel vaccine, currently in clinical trials (ClinicalTrials.gov Identifier: NCT04784767), against VOCs. SpFN-ALFQ, has been previously demonstrated to be highly efficacious against the WA1 strain of SARS-CoV-2 in nonhuman primate and murine models^15,16,18^. In this study, sera from hamsters that were immunized at 10 μg and 0.2 μg of SpFN adjuvanted with fixed concentration of ALFQ as 1- and 2-dose regimens demonstrated dose-dependent binding, neutralizing, and hACE2 inhibiting antibody activity, with the highest responses seen in the 2-dose regimens. The strongest responses against WA1, B.1.1.7 and B.1.351 were observed in the 10 μg group using a 2-dose regimen, with similar binding responses observed between WA1 and B.1.1.7, that were reduced slightly against B.1.351 in the 10 μg, 2-dose regimen but more substantially in the 1-dose regimens. Humoral immunity as assessed by binding antibodies was largely consistent at study weeks 6 and 8, with a sustained, albeit trend toward decreased responses, at the time of challenge (week 11). This was particularly observed in the BLI data as opposed to detection by ELISA, which may be due, in part, to the kinetic nature of the assay. Opsonizing IgG responses, shown to be associated with protection against SARS-CoV-2 in small-animal models, were also elicited^28–30^. Additionally, prior studies have demonstrated that vaccine-induced antibody Fc-mediated functions are also associated with protection against SARS-CoV-2^31–33^. Here, we demonstrated that the SpFN-ALFQ vaccine was able to elicit high IgG opsonization against both wild type and VOCs in hamsters, suggesting that SpFN-ALFQ vaccine-induced antibodies that could leverage Fc-mediated functions associated with protection from SARS-CoV-2 VOCs to include B.1.351. Of note, we observed strong neutralizing responses in the 10 μg, 2-dose regimen groups that were similar across WA1, B.1.1.7 and B.1.351 variants. This is particularly encouraging as many studies have demonstrated decreased neutralizing antibody responses of convalescent or vaccinee sera, in particular against B.1.35^4,5,11,14,25^. hACE2-RBD binding inhibition levels mirrored overall humoral responses, and similar to the binding and neutralizing antibody responses peaked between weeks 6 and 8, with a sustained albeit trend toward decreased response by week 11. The hACE2-RBD assay has functional implications for cellular entry and in conjunction with the binding and neutralization assays, will be critical to utilize in future studies to understand the critical correlates of protection conferred during SpFN-ALFQ vaccination in SGH and NHP models.

As expected from the humoral responses, mitigation of body weight loss was also observed in vaccinated animals, following SARS-CoV-2 VOC challenge, most dramatically in the 2-dose vaccinated animals. Hamsters were challenged with sufficient B.1.1.7 or B.1.351 concentrations, targeted to result in a 10–15% loss of body weight in control animals. In the 2-dose regimen in both the 10 μg and 0.2 μg groups for B.1.1.7, loss of bodyweight was dramatically reduced, from ~12% observed in the PBS control animals to between 2–3% in the vaccinated groups. Notably, a single vaccination using 10 μg of SpFN-ALFQ was also sufficient to confer similar protection, with even the 0.2 μg conferring intermediate levels of protection after a single immunization. In the case of B.1.351, protection in the 2-dose regimen had a similar range of protection, from the ~13% bodyweight loss observed in the PBS treated animals to 3–4%. Vaccine 1-dose regimens were less protective than observed for B.1.1.7 challenge, but still conferred intermediate protection against B.1.351 challenge. These results are highly encouraging, showing potential protective efficacy of the SpFN-ALFQ vaccine against clinical disease caused by VOC to include B.1.351, particularly using a 2-dose regimen.

Following the challenge phase of the study, assessment of viral loads by TCID50 in lung tissues and nasal turbinate demonstrated clear protection from challenge with either B.1.1.7 or B.1.351, with complete elimination of recoverable virus detected in the majority of animals, regardless of vaccine regimen, by day 6 post challenge. Adjunctive analysis of oral swab viral load, either total RNA or subgenomic mRNA, products of discontinuous SARS-CoV-2 transcription only seen during active viral replication, confirmed the lung and nasal turbinate tissue viral culture data in showing virologic evidence of SpFN-ALFQ protection with either B.1.1.7 or B.1.351 challenge.

A key question that this study was designed to address through the utilization of SGH as a pathogenic model of SARS-CoV-2 disease, was whether SpFN-ALFQ had an impact on lung pathology developed during acute infection with B.1.1.7 or B.1.351. Viral challenge doses of B.1.1.7 and B.1.351 utilized in this study gave nearly equivalent pathology in unvaccinated (PBS) control animals, with significant type II pneumocyte hyperplasia and cellular infiltrate occurring within the lung on H&E, and viral staining observed by detection of viral nucleocapsid (N) protein by immunohistochemistry. For B.1.1.7 challenged animals, pathology was dramatically reduced in both the 10 μg and 0.2 μg 2-dose regimens. In all, 1-dose 10 μg and 0.2 μg regimens conferred intermediate levels of protection, with wider variability in individual pathology within individuals. In the B.1.351 challenged animals, the 10 μg 2-dose regimen also demonstrated decreases in pathology compared with the cognate PBS control, with intermediate levels of protection conferred to the 0.2 μg groups. Taken together, SpFN-ALFQ generated robust binding antibody and neutralizing antibody responses in SGH against WA1, B.1.1.7 and B.1.351 SARS-CoV-2 variants. It was also protective in intranasal challenge with the B.1.1.7 and B.1.351 variants as assessed by body weight preservation, lung tissue viral load, oral fluid total and sgmRNA, and standard histopathological and immunohistochemical analysis of lung tissues at necropsy.

Publicly available preprint reports have recently shown that the vaccines being marketed by AstraZeneca, ChAdOx1 nCoV-19 (AZD1222), and Moderna, mRNA-1273, show protection against B.1.351 challenge in hamster and rhesus models, respectively^25,34^. Another preprint describes protection from B.1.351 challenge in human ACE2 transgenic mice vaccinated with the CureVac mRNA vaccine, CVnCoV^35^. Continued molecular epidemiologic analysis of ongoing SARS-CoV-2 infections with concomitant assessment of the impact of emerging VOCs on current and next-generation SARS-CoV-2 vaccines will be essential for achieving and maintaining pandemic control, and underpins the scientific approach to both pan-SARS-CoV-2 and pan-coronavirus vaccine research and development. To this end, SpFN-ALFQ is now being evaluated in a first in human phase I clinical trial as the first step towards broader application to the betacoronavirus genus and the entire family of coronaviruses.

## Methods

### Transfection, expression, and purification of SpFN 1B-06-PL

Expi293 cells (Gibco, Cat No. A14527) were maintained and passaged as per manufacturer’s guidelines in Expi293 Expression Media (Gibco, Cat No. A1435101) at 37 °C, 8% CO_2_, 120 RPM, ≥60% RH. Briefly, the transfection reaction consisted of 1 mg of purified, cGMP sourced plasmid DNA (Aldevron, pCoV 1B-06-PL) plus 3 mL of Turbo 293 Transfection Reagent (Speed Biosystems, Cat No. PXX1001) mixed in 1X PBS per liter of transfected cells. The reaction was incubated at 20–25 °C for 15 min and 125 mL/flask was rapidly and aseptically transferred to 16 single use 3 L Erlenmeyer shake vented flasks (Corning, Cat no. 431252) each containing 1.125 L of Expi293 cells passaged at a density of 2.0 × 10^6^ cells/ml on the day of transfection. After the addition of the transfection reaction, the transfected cells were incubated at 34 °C, 8% CO_2_, 120 RPM, ≥60% RH for 5 days. The expressed product was collected, clarified by double centrifugation at 3700×*g*, 10 °C, for 30 min, followed by depth filtration (0.65 uM + 0.45 uM, Sartorius, Sartobran P, Cat No. 5235306D0-SO-V) and stored at 2–8 °C for further downstream processing.

SpFN 1B-06 PL was purified, concentrated, and dialyzed against 50 mM Tris, 50 mM NaCl, pH 8.0 solution. Briefly, 16L of clarified expression product was initially concentrated 6-fold using a tangential flow filtration module (500 kD MW Cutoff, mPES MiniKros, Cat No.N04-500-05). The concentrate was treated with Benzonase (EMD Millipore, Cat No.EM1.01695.0001) for 120 min at 22 °C before conducting a buffer exchange into 50 mM Tris, 50 mM NaCl, pH 7.915. The material was loaded onto a 4.4 × 14 cm Fractogel DEAE (M) Column (EMD Millipore, Cat No.1168835000) with a bed volume of 212 mL. SpFN 1B-06 PL bound to the column and was eluted with 50 mM Tris Base, 200 mM NaCl, pH 8.018, 21.55 mS/cm. Following this, a 44 mL Capto Core 400 column (Cytiva, Cat No.17372403) was used as a polishing step to remove any potential lower MW contaminants. A final dialysis step was performed to place the product into the final formulation buffer using a 300 kD MWCO UF cartridge (Repligen mPES MiniKros, Cat No. S02-E300-05). The final purified SpFN 1B-06 PL recovery yielded 55.47 mg in a volume of 215 mL, approximately a 3.47 mg/L yield from the cell expansion and growth.

### Viral stock propagation and preparation

B.1.1.7 viral stocks were generated from seed stock (USA/CA_CDC_5574/2020), obtained from BEI resources (Cat# NR-54011, Lot# 70041598) and expanded in Calu-3 cells (incubated at 37 °C for 3 days). The viral stock lot used for this study (Lot# 012921-1230) was titrated in Vero-TMPRSS2 cells, with viral titers of 1.375 × 10^6^ PFU/mL. This stock was used undiluted for viral challenge with 100 μL intranasally, for a final challenge concentration of 1.375 × 10^5^ PFU/mL per animal.

B.1.351 viral stocks were propagated and characterized by deep sequencing^34^. Briefly, hCoV-19/USA/MD-HP01542/2021 (B.1.1.351) was derived from the seed stock (Lot# MD-HP JHU P2) and propagated in in VeroE6-TMPRSS2 cells. The viral titer of the B.1.351 stock used for this study was 3 × 10^7^ PFU/ml in VeroE6-TMPRSS2 cells, diluted 1:100 in PBS for a challenge dose of 3 × 10^4^ PFU in 100 μL per animal.

### Syrian golden hamster immunizations

Male and female Syrian golden hamsters (6–8 week-old, *n* = 55) were acquired from Charles River Laboratories and housed at Bioqual, Inc., for the duration of the study. Following one week of acclimatization, animals were immunized intramuscularly in caudal thighs with PBS (control) or SpFN immunogen of differing concentrations (10 μg or 0.2 μg) pre-formulated with a fixed concentration of ALFQ (20 μg of 3D-PHAD (monophosphoryl 3-deacyl lipid A (synthetic)) and 10 μg of QS21)^36^. The design, production, stability, and initial characterization of SpFN adjuvanted with ALFQ was previously reported^18^. Briefly, the immunogen is based on the Spike protein of the WA1 SARS-CoV-2 variant with S-2P amino acid modifications expressed as a fusion protein with H. pylori ferritin in mammalian cells that self-assembles into an ordered nanoparticle each of which presents 8 Spike trimers. SpFN was formulated with ALFQ prior to administration. SpFN was produced at the WRAIR Pilot Bioproduction Facility in October 2020 as an engineering batch and stored at 4 °C for 3–4 months for the 1- and 2-dose regimens, respectively. Boosting immunizations were injected contralaterally from the prime four weeks following the prime. In-life blood sampling was conducted by retro-orbital bleeds. Maximum blood collections were determined based on animal weight and frequency of collection, in consultation with veterinary guidance. Following collections, animals were monitored until fully recovered from the anesthetic and the procedure.

### SARS-CoV-2 Syrian golden hamster challenge

Animals were challenged with SARS-CoV-2 seven weeks following the boost or single immunization. Animals were anesthetized with ketamine/xylazine and challenged by intranasal inoculation of 50 µL virus in each nostril in a drop-wise manner (100 µL/hamster). Challenge dose was pre-determined for each VOC viral stock to achieve comparable clinical disease as manifested by body weight loss of 10–15%. B.1.1.7 virus was administered at a challenge dose of 1.375 × 10^5^. B.1.351 virus was administered at a challenge dose of 3 × 10^4^ PFU per hamster. Following viral challenge, all animals were weighed and observed twice daily for clinical signs (ruffled fur, hunched posture, behavior, etc.), with euthanasia criteria of 20% loss of pre-challenge body weight or becoming moribund. At study termination 6 DPC, all animals were terminally anesthetized by ketamine/xylazine, followed by exsanguination by cardiac puncture (for terminal blood collection) and euthanasia. Tissues were collected for use in downstream virologic, immunologic, molecular and histopathology studies. Animals were housed in BLS-2 during the vaccination phase and BSL-3 facilities during the challenge phase.

### Enzyme linked immunosorbent assay

In total, 96-well Immulon “U” Bottom plates were coated with 1 μg/mL of RBD or S protein (S-2P) antigen in Dulbecco’s PBS, pH 7.4. Plates were incubated at 4 °C overnight and blocked with blocking buffer (PBS containing 0.5% Casein and 0.5% BSA, pH 7.4), at room temperature (RT) for 2 h. Individual serum samples were serially diluted 2-fold in blocking buffer and added to triplicate wells and the plates were incubated for 1 h at RT. The plates were washed with PBS containing 0.1% Tween 20, pH 7.4, followed by the addition of horseradish peroxidase (HRP)-conjugated goat anti-Hamster IgG (H + L) (1:2000 dilution) (Cat. 6060-05, SouthernBiotech) for an hour at RT. The HRP substrate, 2,2′-Azinobis [3-ethylbenzothiazoline-6-sulfonic acid]-diammonium salt (ABTS) (KPL) was added to the plates for 1 h at RT. The reaction was stopped by the addition of 1% SDS per well and the absorbance (A) was measured at 405 nm (A405) using an ELISA reader Spectramax (Molecular Devices, San Jose, CA) within 30 min of stopping the reaction. The results are expressed as end point titers, defined as the reciprocal dilution that gives an absorbance value that equals twice the background value (antigen-coated wells that did not contain the test sera, but had all other components added).

### Biolayer interferometry RBD binding assay

All biosensors were hydrated in PBS prior to use. All assay steps were performed at 30 °C with agitation set at 1000 rpm in the Octet RED96 instrument (FortéBio). HIS1K biosensors (FortéBio) were equilibrated in assay buffer (PBS) for 30 s before loading of His-tagged SARS-CoV-2 WA1 RBD or VOC RBDs B1.1.7, and B.1.351 (30 μg/mL diluted in PBS) for 120 s. Immobilized RBD proteins were then dipped in hamster sera (100x dilution with PBS) for 180 s followed by dissociation for 60 s. Binding response values were recorded at 180 s. SARS-CoV-2 RBD constructs (residues 331–527), modified to incorporate a N-terminal hexa-histadine tag (for purification), were derived from the Wuhan-Hu-1 strain genome sequence (GenBank MN9089473) and synthesized and subcloned into a CMVR plasmid by Genscript. RBD with VOC point mutations were generated using a modified QuikChange site-directed mutagenesis protocol (Agilent). The constructs resulting from site-directed mutagenesis were amplified and isolated from E. coli Stbl3 or Top10 cells. Large-scale DNA isolation was performed using either endo free Maxiprep, Megaprep or Gigaprep kits (Qiagen). All expression plasmids were transiently transfected into Expi293F cells (Thermo Fisher Scientific) using ExpiFectamine 293 transfection reagent (Thermo Fisher Scientific). Cells were grown in polycarbonate baffled shaker flasks at 34 °C and 8% CO_2_ at 120 rpm. Cells were harvested 5–6 days post-transfection via centrifugation at 3500×*g* for 30 min. Culture supernatants were filtered with a 0.22-µm filter and stored at 4 °C prior to purification. His-tagged RBD proteins were purified using Ni-NTA affinity chromatography, with 1 mL Ni-NTA resin (Thermo Scientific) used to purify protein from 1 L of expression supernatant. Ni-NTA resin was equilibrated with 5 column volumes (CV) of phosphate buffered saline (PBS) (pH 7.4) followed by supernatant loading 2x at 4 °C. Unbound protein was removed by washing with 200 CV of PBS, followed by 50 CV 10 mM imidazole in PBS. Bound protein was eluted with 220 mM imidazole in PBS. All proteins were further purified by size-exclusion chromatography using a 16/60 Superdex-200 purification column. Purification purity for all the proteins was assessed by SDS-PAGE.

### Biolayer Interferometry hACE2 competition assay

All biosensors were hydrated in PBS prior to use. All assay steps were performed at 30 °C with agitation set at 1000 rpm in the Octet RED96 instrument (FortéBio). SARS-CoV-2 RBD - hACE2 competition assays were carried out as follows. SARS-CoV-2 RBD (WA1 strain, 30 μg/ml diluted in PBS) was immobilized on HIS1K biosensors (FortéBio) for 180 s followed by baseline equilibration for 30 s. Serum binding was allowed to occur for 180 s followed by baseline equilibration (30 s). Recombinant hACE2 protein (30 μg/ml) was then allowed to bind for 120 s. Percent inhibition (PI) of hACE2 binding to the RBD by serum was determined using the equation: PI = 100 − ((hACE2 binding in the presence of mouse serum/hACE2 binding in the absence of mouse serum) × 100).

### IgG opsonization assays

SARS-CoV-2 S-expressing expi293F cells were generated by transfection with linearized plasmid (pcDNA3.1) encoding codon-optimized full-length SARS-CoV-2 S protein matching the amino acid sequence of the IL-CDC-IL1/2020 isolate (GenBank ACC# MN988713), the B.1.1.7 isolate^9^, or the B.1.351 isolate^37^. Stable transfectants were single-cell sorted and selected to obtain a high-level Spike surface expressing clone (293F-Spike-S2A). 293F-Spike-S2A cells were incubated with 100 μl of plasma diluted 100-fold in RPMI containing 10% FBS (R10) for 30 min at 37 °C. Cells were washed three times and stained with a goat anti-hamster IgG (H + L) Alexa Fluor 488 diluted 1:250 (Cat A-21110, ThermoFisher Scientific). Cells were then fixed with 4% formaldehyde solution and fluorescence was evaluated on a LSRII analytic cytometer (BD Bioscience).

### SARS-CoV-1 and SARS-CoV-2 pseudovirus neutralization assay

Pseudovirions were produced by co-transfection of HEK293T/17 cells with either the SARS-CoV-1 (Sino 1–11, GenBank # AY485277) or SARS-CoV-2 (WA1/2020 GenBank # MT246667) S expression plasmid and an HIV-1 pNL4-3 luciferase reporter plasmid (pNL4-3.Luc.R-E-, NIH AIDS Reagent Program). The S expression plasmid sequences for SARS-CoV-2 and SARS-CoV-1 were codon optimized and modified to remove an 18 amino acid endoplasmic reticulum retention signal in the cytoplasmic tail in the case of SARS-CoV-2, and a 28 amino acid deletion in the cytoplasmic tail in the case of SARS-CoV-1 to improve S incorporation into pseudovirions and improve infectivity. S expression plasmids for SARS-CoV-2 VOC were similarly codon optimized, modified, and included the following mutations: B.1.1.7 (69-70del, Y144del, N501Y, A570D, D614G, P681H, T718I, S982A, D1118H), B.1.351 (L18F, D80A, D215G, 241-243del, K417N, E484K, N501Y, D614G, A701V, E1195Q). Virions pseudotyped with the vesicular stomatitis virus (VSV) G protein were used as a non-specific control. Infectivity and neutralization titers were determined using ACE2-expressing HEK293 target cells (Integral Molecular). Test sera were diluted 1:40 in cell culture medium and serially diluted; then 25 μL/well was added to a white 96-well plate. An equal volume of diluted SARS-CoV-2 PSV was added to each well and plates were incubated for 1 h at 37 °C. Target cells were added to each well (40,000 cells/ well) and plates were incubated for an additional 48 h. Relative light units (RLU) were measured with the EnVision Multimode Plate Reader (Perkin Elmer, Waltham, MA) using the Bright-Glo Luciferase Assay System (Promega, Madison, WI). Neutralization dose–response curves were fitted by nonlinear regression using the LabKey Server. Final titers are reported as the reciprocal of the dilution of serum necessary to achieve 50% (ID50, 50% inhibitory dose). Assay equivalency for SARS-CoV-2 was established by participation in the SARS-CoV-2 Neutralizing Assay Concordance Survey (SNACS) run by the Virology Quality Assurance Program and External Quality Assurance Program Oversite Laboratory (EQAPOL) at the Duke Human Vaccine Institute, sponsored through programs supported by the National Institute of Allergy and Infectious Diseases, Division of AIDS.

### Oral cavity viral RNA measurements

The oral cavity was swabbed with a sterile flocked swab that was immediately placed into a cryovial with 1 mL PBS. Vials were snap-frozen on dry ice and stored at −80 °C until testing. Viral RNA was extracted from 200 μl of oral swab material using the Qiagen EZ1 DSP Virus kit on the automated EZ1 XL Advance instrument (Qiagen, Valencia, CA). Real-time quantitative reverse transcription – polymerase chain reactions (RT-qPCR) were performed on the 7500 Dx Fast thermal cycler (Thermo Fisher Scientific, Life Technologies, Carlsbad, CA). SARS-CoV-2 specific forward (*TCGTGGTATTCTTGCTAG*) and reverse primers (*GAAGGTTTTACAAGACTCAC*) and probe (*FAM -ACACTAGCCATCCTTACTGCG-BHQ1*) targeting the E gene encoding the envelope protein were used for amplification of the viral RNA. Amplification of the sgmRNA was achieved using the Leader TRS sequence specific forward primer (*CGATCTCTTGTAGATCTGTTCTC*) and the same E reverse primer and probe as used to quantify total viral RNA^16^. A synthetic RNA for subgenomic E was used as a calibrator. Final results were reported in copies/ml.

### Tissue viral burden by TCID50

The infectious titer determination from lungs and nasal turbinates was obtained by performing a TCID50 assay. Vero TMPRSS2 cells were plated at 25,000 cells per well in DMEM supplemented with 10% FBS and gentamicin. Cells were incubated at 37 °C, 5.0% CO2. When cells achieved 80–100% confluency, the media was aspirated and replaced with 180 μL of DMEM containing 2% FBS and gentamicin. 0.10–0.20 mg sections of the right lobe of the lung and the nasal turbinates were collected at necropsy, snap frozen, and stored at −80 °C until processing. Frozen tissue was placed in 15 mL conical tube on wet ice containing 0.5 mL media and homogenized 10–30 s (Probe, Omni International: 32750H). The tissue homogenate was spun to remove debris at 2000×*g*, 4 °C for 10 min. The supernatant was then passed through a strainer (Pluriselect: Cat No. 43-10100-40) into the original vial and kept on wet ice. From the strained supernatant, 20 μL aliquots were tested in quadruplicate in a 96-well plate format. The top row of the 96-well plate was mixed five times and serially diluted by pipette transfer of 20 μL, representing 10-fold dilutions. Pipette tips were disposed of between each row and mixing was repeated until the last row on the plate. After incubation for 4 days, wells were visually inspected for cytopathic effects (CPE) scored as CPE minus (−) where non-infected wells have a clear confluent cell layer, or CPE plus (+), where rounding of infected cells is observed. Positive controls were utilized for optimal assay performance, where the TCID50 tested within 2-fold of the expected value. TCID50 of all samples were calculated using the Read-Muench formula.

### Histology and immunohistochemistry

Lungs from 6 DPC were insufflated and perfused with 10% neutral-buffered formalin. Three tissue sections from each of the left lung lobes were used to evaluate the lung pathology. Sections were processed routinely into paraffin wax, then sectioned at 5 µm, and resulting slides were stained with hematoxylin and eosin. Immunohistochemistry (IHC) on formalin fixed paraffin embedded tissue sections was performed using the Dako Envision system (Dako Agilent Pathology Solutions, Carpinteria, CA, USA). Briefly, after deparaffinization, peroxidase blocking, and antigen retrieval, sections were stained with an anti-SARS-CoV/SARS-COV-2 nucleocapsid (N) protein rabbit monoclonal antibody (Cat. 40143-R001, Sino Biological, Chesterbrook, PA, USA) at a dilution of 1:6000 and incubated at RT for 45 min. Sections were rinsed and stained with peroxidase-labeled polymer (secondary antibody) for 30 min. Slides were rinsed and a brown chromogenic substrate 3,3′ Diaminobenzidine (DAB) solution (Dako Agilent Pathology Solutions) was applied for 8 min. Slides were rinsed, counterstained with hematoxylin, and rinsed. The sections were dehydrated, cleared with Xyless II, and cover-slipped. All tissue slides were evaluated by a board-certified veterinary anatomic pathologist blinded to study group allocations. Semi-quantitative scoring of pulmonary pathology was performed, with grading of intra-alveolar edema, type II pneumocyte hyperplasia, mononuclear cellular infiltrates, polymorphonuclear cellular infiltrates, alveolar histiocytosis, alveolar necrosis, bronchioalveolar epithelial degeneration, bronchiolar epithelial hyperplasia, and interstitial collagenous deposition. Each finding was scored as follows: 0 – absent, 1 – minimal (<10% of tissue section affected); 2 – mild (11–25% of tissue section affected); 3 – moderate (26–50% of tissue section affected); 4 – marked (51–75% affected); and 5 – severe (>75% of tissue section affected). IHC sections were examined at ×400 magnification and evaluated for the number of immunopositive cells per slide.

### Ethical statement

All animal in vivo procedures were carried out in accordance with institutional, local, state, and national guidelines and laws governing research in animals including the Animal Welfare Act. Animal protocols and procedures complied with all relevant ethical regulations for animal testing and research and were reviewed and approved by the Animal Care and Use Committee of the US Army Medical Research and Development Command (USAMRDC) Animal Care and Use Review Office as well as the Institutional Animal Care and Use Committee of Bioqual, Inc. (protocol number 20–144), including ethical approval. Bioqual, Inc., and the USAMRDC are accredited by the Association for Assessment and Accreditation of Laboratory Animal Care and are in full compliance with the Animal Welfare Act and Public Health Service Policy on Humane Care and Use of Laboratory Animals. Oversight of all research was approved and conducted by the WRAIR Institutional Biological Safety Committee.

### Statistical analysis

All statistical analyses were performed using GraphPad Prism version 8 software. All statistical tests shown were performed using the Kruskal-Wallis test with Dunn’s correction, comparing all variables (multiple comparison analysis, not against a standard control). Actual *p*-values for each comparison annotated for statistically relevant values, or for values near statistical significance. All other not statistically or biologically relevant values reported as not-significant (ns). Comparisons to PBS control group are shown directly above each vaccine treatment condition (gray), with inter/intra vaccine regimen comparisons indicated by a solid line between groups, with the statistical result directly above (black). Tests were performed with one-sided design and assays were done in replicate.

### Reporting summary

Further information on research design is available in the Nature Research Reporting Summary linked to this article.

## Supplementary information

Supplementary Information

Reporting Summary

## Data Availability

The datasets generated during and/or analyzed during the current study are available from the corresponding author on reasonable request.

## References

[1] Craven, J. (Regulatory Affairs Professionals Society, 2021). https://www.raps.org/news-and-articles/news-articles/2020/3/covid-19-vaccine-tracker.

[2] Hu B, Guo H, Zhou P, Shi ZL (2021). Characteristics of SARS-CoV-2 and COVID-19. Nat. Rev. Microbiol..

[3] CDC. *SARS-CoV-2 Variant Classifications and Definitions*, https://www.cdc.gov/coronavirus/2019-ncov/cases-updates/variant-surveillance/variant-info.html#Concern (2021).

[4] Wang P (2021). Antibody resistance of SARS-CoV-2 variants B.1.351 and B.1.1.7. Nature.

[5] Wibmer CK (2021). SARS-CoV-2 501Y.V2 escapes neutralization by South African COVID-19 donor plasma. Nat. Med..

[6] Wu, K. et al. mRNA-1273 vaccine induces neutralizing antibodies against spike mutants from global SARS-CoV-2 variants. Preprint at: 10.1101/2021.01.25.427948 (2021).

[7] Galloway SE (2021). Emergence of SARS-CoV-2 B.1.1.7 Lineage - United States, December 29, 2020–January 12, 2021. MMWR Morb. Mortal. Wkly Rep..

[8] Tegally H (2021). Detection of a SARS-CoV-2 variant of concern in South Africa. Nature.

[9] Rambaut, A. et al. on behalf of COVID-19 Genomics Consortium UK (CoG-UK). *Preliminary Genomic Characterisation Of An Emergent Sars-cov-2 Lineage In The UK Defined By A Novel Set Of Spike Mutations*. https://virological.org/t/preliminary-genomic-characterisation-of-an-emergent-sars-cov-2-lineage-in-the-uk-defined-by-a-novel-set-of-spike-mutations/563/1 (2020).

[10] Hacisuleyman E (2021). Vaccine breakthrough infections with SARS-CoV-2 variants. New Engl. J. Med..

[11] Ho, D. et al. Increased resistance of SARS-CoV-2 variants B.1.351 and B.1.1.7 to antibody neutralization. *Res Sq.*10.21203/rs.3.rs-155394/v1.

[12] Hu J (2021). Emerging SARS-CoV-2 variants reduce neutralization sensitivity to convalescent sera and monoclonal antibodies. Cell Mol. Immunol..

[13] Cohen, J. *Science Magazine.* American Association for the Advancement of Science. (2021).

[14] Garcia-Beltran WF (2021). Multiple SARS-CoV-2 variants escape neutralization by vaccine-induced humoral immunity. Cell.

[15] Alving CR, Peachman KK, Matyas GR, Rao M, Beck Z (2020). Army Liposome Formulation (ALF) family of vaccine adjuvants. Expert Rev. Vaccines.

[16] Joyce, M. G. et al. Efficacy of a broadly neutralizing SARS-CoV-2 ferritin nanoparticle vaccine in nonhuman primates. Preprint at: 10.1101/2021.03.24.436523.

[17] King HAD (2021). Efficacy and breadth of adjuvanted SARS-CoV-2 receptor-binding domain nanoparticle vaccine in macaques.. Proc. Natl. Acad. Sci. U S A.

[18] Joyce, M. G. et al. SARS-CoV-2 ferritin nanoparticle vaccines elicit broad SARS coronavirus immunogenicity. Preprint at: 10.1101/2021.05.09.443331. 10.1016/j.celrep.2021.110143 PMC8651551 34919799

[19] Chan JF (2020). Simulation of the Clinical and Pathological Manifestations of Coronavirus Disease 2019 (COVID-19) in a Golden Syrian hamster model: implications for disease pathogenesis and transmissibility. Clin. Infect. Dis..

[20] Sia SF (2020). Pathogenesis and transmission of SARS-CoV-2 in golden hamsters. Nature.

[21] Imai M (2020). Syrian hamsters as a small animal model for SARS-CoV-2 infection and countermeasure development. Proc. Natl Acad. Sci. USA.

[22] Tostanoski LH (2020). Ad26 vaccine protects against SARS-CoV-2 severe clinical disease in hamsters. Nat. Med..

[23] Munoz-Fontela C (2020). Animal models for COVID-19. Nature.

[24] Hoffmann M (2020). SARS-CoV-2 cell entry depends on ACE2 and TMPRSS2 and is blocked by a clinically proven protease inhibitor. Cell.

[25] Fischer RJ (2021). ChAdOx1 nCoV-19 (AZD1222) protects hamsters against SARS-CoV-2 B.1.351 and B.1.1.7 disease. Nat. Commun..

[26] CDC. COVID Data Tracker. https://covid.cdc.gov/covid-data-tracker/?CDC_AA_refVal=https%3A%2F%2Fwww.cdc.gov%2Fcoronavirus%2F2019-ncov%2012Fcases-updates%2012Fvariant-proportions.html#variant-proportions (2021).

[27] Karim, F. M. et al. Persistent SARS-CoV-2 infection and intra-host evolution in association with advanced HIV infection. Preprint at: 10.1101/2021.06.03.21258228.

[28] Schafer, A. et al. Antibody potency, effector function, and combinations in protection and therapy for SARS-CoV-2 infection in vivo. *J. Exp. Med.*10.1084/jem.20201993 (2021). 10.1084/jem.20201993 PMC7673958 33211088

[29] Suryadevara N (2021). Neutralizing and protective human monoclonal antibodies recognizing the N-terminal domain of the SARS-CoV-2 spike protein. Cell.

[30] Winkler ES (2021). Human neutralizing antibodies against SARS-CoV-2 require intact Fc effector functions for optimal therapeutic protection. Cell.

[31] Mercado NB (2021). Publisher correction: single-shot Ad26 vaccine protects against SARS-CoV-2 in rhesus macaques. Nature.

[32] Mercado NB (2020). Single-shot Ad26 vaccine protects against SARS-CoV-2 in rhesus macaques. Nature.

[33] Yu J (2020). DNA vaccine protection against SARS-CoV-2 in rhesus macaques. Science.

[34] Corbett KS (2020). Evaluation of the mRNA-1273 vaccine against SARS-CoV-2 in nonhuman primates. New Engl. J. Med..

[35] Hoffmann DC (2021). CVnCoV protects human ACE2 transgenic mice from ancestral B BavPat1 and emerging B.1.351 SARS-CoV-2. Nat. Commun..

[36] Carmen, J. S. et al. A spike-ferritin nanoparticle vaccine induces robust innate immune activity and drives polyfunctional SARS-CoV-2-specific T cells. Preprint at: 10.1101/2021.04.28.441763. 10.1038/s41541-021-00414-4 PMC8668928 34903722

[37] Tegally H (2021). Emergence and rapid spread of a new severe acute respiratory syndrome-related coronavirus 2 (SARS-CoV-2) lineage with multiple spike mutations in South Africa. Nature.

