# Supplementary material for: A SARS-CoV-2 spike ferritin nanoparticle vaccine protects hamsters against Alpha and Beta virus variant challenge
Source: NPJ Vaccines. 2021 Oct 28;6:129. doi: 10.1038/s41541-021-00392-7 (PMC8553838; doi:10.1038/s41541-021-00392-7)
Supplement: Supplementary file 1 — Supplementary Information [file 41541_2021_392_MOESM1_ESM.pdf]

Supplementary Figure 1

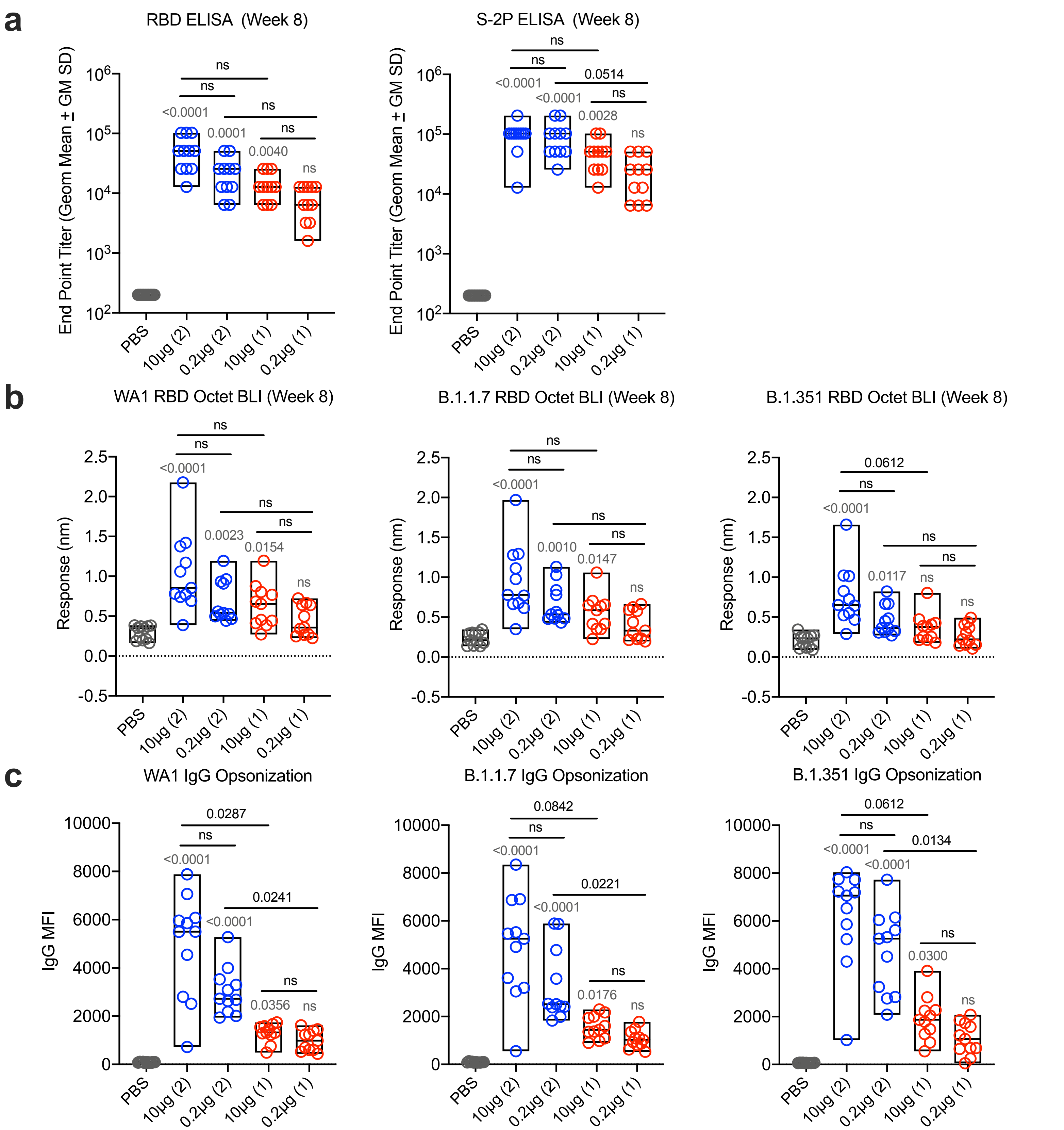

Supplementary Figure 1. Additional antibody responses following SpFN-ALFQ immunization. **a,** ELISA was performed using either WA1 derived Receptor Binding Domain (RBD) or S-2P Spike proteins from sera taken at week 8. The vaccine regimens are indicated on the x-axis by phosphate buffered saline (PBS) control or SpFN concentration with the number of vaccinations in the regimen given parenthetically and by color code (blue, 2-dose, red, 1-dose). Endpoint titers are given on the y-axis as geometric mean titers. **b,** Octet Biolayer Interferometry (BLI) responses against the WA1, B.1.1.7, and B.1.351 sequences of the RBD are given for the vaccination regimens as in **a** from sera collected at week 8. BLI responses are given in nanometers (nm) on the y-axis. **c** IgG opsonization as measured by binding to SARS-CoV-2 Spike protein expressing expi293F cells subsequently stained by fluorescently tagged goat anti-hamster IgG and detected by flow cytometry. Fluorescence is given as mean fluorescence intensity (MFI) on the y-axis. Box plot bounds depict the standard deviation and the center line the median value. P-values for SpFN-ALFQ vaccination groups compared with PBS control are given just above the boxes in light grey while inter- and intra-regimen p-values are given above the boxes in black. ns, not significant ($p > 0.05$).

Supplementary Figure 2

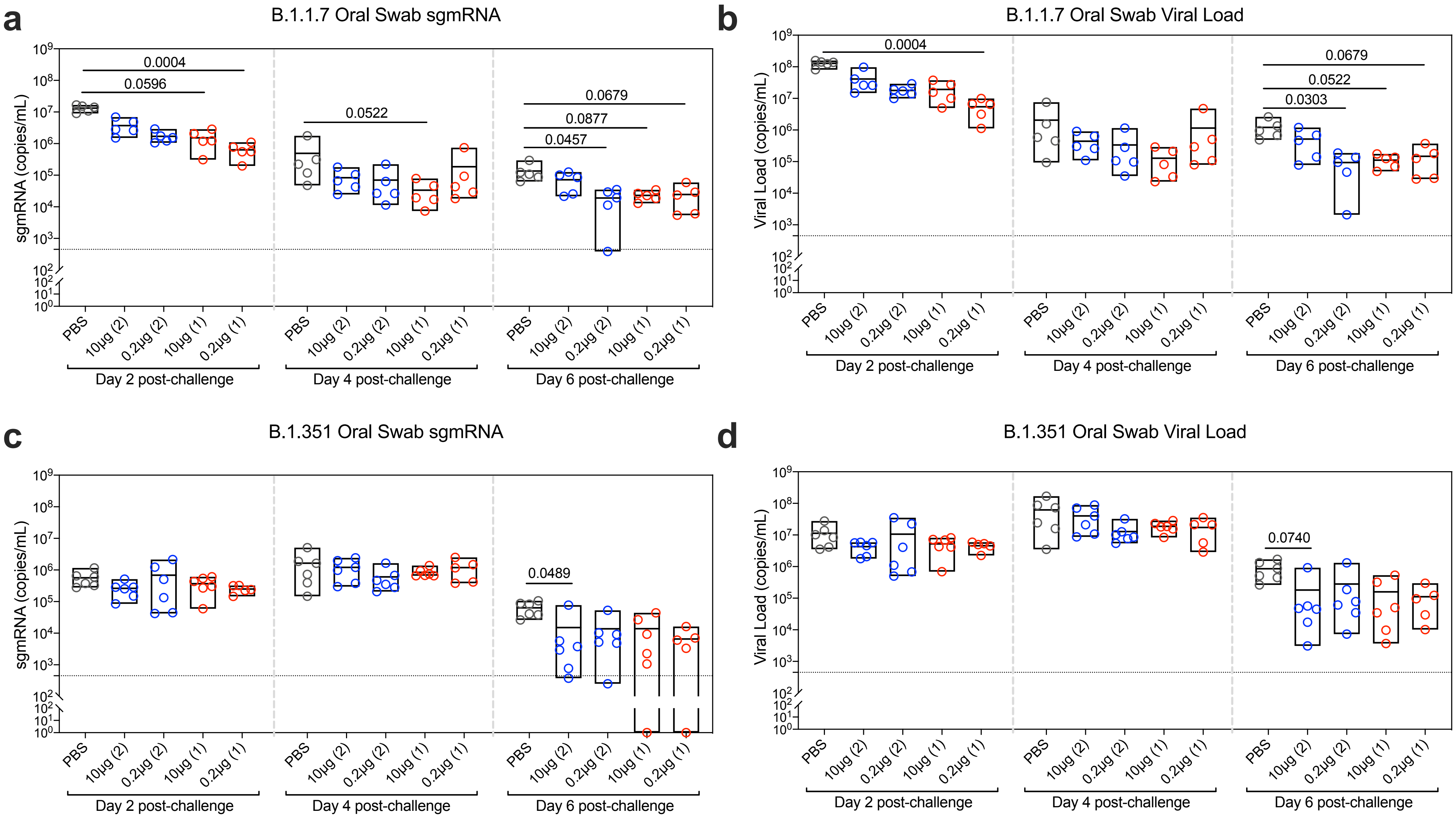

Supplementary Figure 2. Quantitative SARS-CoV-2 total and subgenomic mRNA (sgmRNA) viral load from oral swabs following challenge. Oral fluid collections were obtained by swabbing hamsters at days 2, 4, and 6 post challenge and submitted to quantitative SARS-CoV-2 total and sgmRNA analysis. Viral load is given on y-axes at copies/mL of oral fluid plotted on a log10 scale. Vaccination regimen groups, stratified by day post-challenge, are given on the x-axes: PBS control, gray circles; 10 g and 0.2 g groups in the 2-dose regimen, blue circles; 10 g and 0.2 g groups in the 1-dose regimen, red circles. **a,** sgmRNA viral load following B.1.1.7 challenge; **b,** total RNA viral load following B.1.1.7 challenge; **c,** sgmRNA viral load following B.1.351 challenge; **d,** total RNA viral load following B.1.351 challenge. Box plot bounds reflect the group minimum and maximum values; center line is the median value for each group. Significant differences between groups are indicated by p-values and horizontal black bars. In all other cases, the p-values were > 0.05.

Supplementary Table 1a

			IP Pathology (# observed/total n)				
**Challenge Strain**	**Vaccine regimen**	**Vaccine dose**	**None**	**Minimal**	**Mild**	**Moderate**	**Marked**
**B.1.1.7**	Prime-boost	PBS				3/5	2/5
		10 µg (2)	3/5	2/5			
		0.2 µg (2)	2/5	3/5			
	Single	10 µg (1)	2/5	1/5	1/5		1/5
		0.2 µg (1)	1/5	1/5		2/5	1/5
**B.1.351**	Prime-boost	PBS				4/6	2/6
		10 µg (2)	4/6	1/6	1/6		
		0.2 µg (2)	1/6	2/6	1/6	1/6	1/6
	Single	10 µg (1)	1/6	3/6	2/6		
		0.2 µg (1)*		3/5	1/5		1/5
*one animal lost to study due to injury unrelated to infection.							

Supplementary Table 1b

			SARS-CoV-2 Viral Antigen per Section (# animals observed/total n)					
**Challenge Strain**	**Vaccine regimen**	**Vaccine dose**	**None**	**Single cell**	**<5 cells**	**<20 cells**	**<40 cells**	**>500 cells**
**B.1.1.7**	Prime-boost	PBS						5/5
		10 µg (2)	3/5	2/5				
		0.2 µg (2)	4/5	1/5				
	Single	10 µg (1)	3/5		1/5	1/5		
		0.2 µg (1)	1/5	1/5	1/5	2/5		
**B.1.351**	Prime-boost	PBS						6/6
		10 µg (2)	5/6			1/6		
		0.2 µg (2)	1/6	5/6				
	Single	10 µg (1)	4/6			2/6		
		0.2 µg (1)*		2/5	2/5		1/5	
*one animal lost to study due to injury unrelated to infection.								

Supplementary Table 1. Semi-quantitative histopathology and IHC scores

Lung tissues were collected at necropsy on day 6 post-challenge, fixed with neutral buffered formalin, and stained with hematoxylin and eosin (H&E) for standard microscopic examination as well as submitted for immunohistochemical (IHC) staining for SARS-CoV-2 nucleocapsid (N) protein. **a,** H&E sections were semi-quantitatively scored on the histopathology present in all lung sections examined (Methods). The overall severity of interstitial pneumonia (IP) present in individual animals in the PBS control and vaccinated groups is given. **b,** IHC sections were examined at 400X magnification and evaluated for the number of immunopositive cells per slide. Distribution of the number of immunopositive cells per section is given per PBS control and 2-dose and 1-dose vaccine groups. The number of vaccinations per vaccine group are given parenthetically.
