# Supplementary material for: A SARS-CoV-2 spike ferritin nanoparticle vaccine protects hamsters against Alpha and Beta virus variant challenge
Source: NPJ Vaccines. 2021 Oct 28;6:129. doi: 10.1038/s41541-021-00392-7 (PMC8553838; doi:10.1038/s41541-021-00392-7)
Supplement: Supplementary file 2 — Reporting Summary [file 41541_2021_392_MOESM2_ESM.pdf]

nature portfolio

Corresponding author(s): DBPR NPJVACCINES-01479-T

Last updated by author(s): Jul 22, 2021

Reporting Summary

Nature Portfolio wishes to improve the reproducibility of the work that we publish. This form provides structure for consistency and transparency in reporting. For further information on Nature Portfolio policies, see our Editorial Policies and the Editorial Policy Checklist.

Statistics

For all statistical analyses, confirm that the following items are present in the figure legend, table legend, main text, or Methods section.

n/a	Confirmed	
☐	☒	The exact sample size (n) for each experimental group/condition, given as a discrete number and unit of measurement
☐	☒	A statement on whether measurements were taken from distinct samples or whether the same sample was measured repeatedly
☐	☒	The statistical test(s) used AND whether they are one- or two-sided<br>*Only common tests should be described solely by name; describe more complex techniques in the Methods section.*
☒	☐	A description of all covariates tested
☒	☐	A description of any assumptions or corrections, such as tests of normality and adjustment for multiple comparisons
☐	☒	A full description of the statistical parameters including central tendency (e.g. means) or other basic estimates (e.g. regression coefficient) AND variation (e.g. standard deviation) or associated estimates of uncertainty (e.g. confidence intervals)
☒	☐	For null hypothesis testing, the test statistic (e.g. F, t, r) with confidence intervals, effect sizes, degrees of freedom and P value noted<br>*Give P values as exact values whenever suitable.*
☒	☐	For Bayesian analysis, information on the choice of priors and Markov chain Monte Carlo settings
☒	☐	For hierarchical and complex designs, identification of the appropriate level for tests and full reporting of outcomes
☒	☐	Estimates of effect sizes (e.g. Cohen's d, Pearson's r), indicating how they were calculated

Our web collection on statistics for biologists contains articles on many of the points above.

Software and code

Policy information about availability of computer code

Data collection	Devices used for data acquisition are enumerated in the Methods sections whose native software was used.
Data analysis	GraphPad Prism version 8 software. Neutralization dose–response curves were fitted by nonlinear regression using the LabKey Server.

For manuscripts utilizing custom algorithms or software that are central to the research but not yet described in published literature, software must be made available to editors and reviewers. We strongly encourage code deposition in a community repository (e.g. GitHub). See the Nature Portfolio guidelines for submitting code & software for further information.

Data

Policy information about availability of data

All manuscripts must include a data availability statement. This statement should provide the following information, where applicable:

- Accession codes, unique identifiers, or web links for publicly available datasets
- A description of any restrictions on data availability
- For clinical datasets or third party data, please ensure that the statement adheres to our policy

The datasets generated during and/or analysed during the current study are available from the corresponding author on reasonable request.

Field-specific reporting

Please select the one below that is the best fit for your research. If you are not sure, read the appropriate sections before making your selection.

☒ Life sciences ☐ Behavioural & social sciences ☐ Ecological, evolutionary & environmental sciences

For a reference copy of the document with all sections, see nature.com/documents/nr-reporting-summary-flat.pdf

Life sciences study design

All studies must disclose on these points even when the disclosure is negative.

Sample size	Sample sizes were based on similar experimental designs by others in the field contemporaneously.
Data exclusions	No data were excluded.
Replication	Diane to fill in.
Randomization	Animals were randomly chosen for study arms.
Blinding	The study team was not blinded to group data. Blinding is uncommonly done in small animal experiments such as these.

Reporting for specific materials, systems and methods

We require information from authors about some types of materials, experimental systems and methods used in many studies. Here, indicate whether each material, system or method listed is relevant to your study. If you are not sure if a list item applies to your research, read the appropriate section before selecting a response.

Materials & experimental systems

n/a	Involved in the study	
☐	☒	Antibodies
☐	☒	Eukaryotic cell lines
☒	☐	Palaeontology and archaeology
☐	☒	Animals and other organisms
☒	☐	Human research participants
☒	☐	Clinical data
☒	☐	Dual use research of concern

Methods

n/a	Involved in the study	
☒	☐	ChIP-seq
☒	☐	Flow cytometry
☒	☐	MRI-based neuroimaging

Antibodies

Antibodies used	anti-SARS-CoV/SARS-COV-2 nucleocapsid (N) protein rabbit monoclonal antibody (#40143-R001, Sino Biological, Chesterbrook, PA, USA)
Validation	Validation done by the use of controls as mentioned in the MS.

Eukaryotic cell lines

Policy information about cell lines

Cell line source(s)	Expi293 cells (Gibco, Cat No. A14527); VeroE6-TMPRSS2 cells; Calu-3 cells
Authentication	Cell lines were not authenticated.
Mycoplasma contamination	Expi293 cells were certified Mycoplasma sp free. This was subsequently tested in GLP testing.
Commonly misidentified lines (See ICLAC register)	None.

Animals and other organisms

Policy information about studies involving animals; ARRIVE guidelines recommended for reporting animal research

Laboratory animals	Male and female Syrian golden hamsters (6-8 week-old, n = 55) were acquired from Charles River Laboratories .

Wild animals	not applicable
Field-collected samples	not applicable
Ethics oversight	All animal in vivo procedures were carried out in accordance with institutional, local, state, and national guidelines and laws governing research in animals including the Animal Welfare Act. Animal protocols and procedures were reviewed and approved by the Animal Care and Use Committee of both the US Army Medical Research and Development Command (USAMRDC) Animal Care and Use Review Office as well as the Institutional Animal Care and Use Committee of Bioqual, Inc. (protocol number 20-144). Bioqual, Inc. and the USAMRDC are accredited by the Association for Assessment and Accreditation of Laboratory Animal Care and are in full compliance with the Animal Welfare Act and Public Health Service Policy on Humane Care and Use of Laboratory Animals. Oversight of all research was approved and conducted by the WRAIR Institutional Biological Safety Committee.

Note that full information on the approval of the study protocol must also be provided in the manuscript.
